# 异基因造血干细胞移植治疗儿童急性髓系白血病（非急性早幼粒细胞白血病）中国专家共识（2022年版）

**DOI:** 10.3760/cma.j.issn.0253-2727.2022.10.002

**Published:** 2022-10

**Authors:** 中华医学会血液学分会干细胞应用学组 中华医学会儿科学分会

儿童急性髓系白血病（acute myeloid leukemia, AML）是儿童时期发病率第二位的白血病，占儿童白血病总数的15％～20％[1]，目前长期生存率为60％～70％[2]–[4]。异基因造血干细胞移植（allo-HSCT）作为巩固治疗，与大剂量阿糖胞苷/蒽环类药物一起在儿童AML治疗中发挥着主要作用。二代测序技术的普及使得儿童AML危险分层得以精准，新型靶向药物的应用及allo-HSCT技术的成熟进一步改善了我国儿童AML患者的长期生存。为了规范我国allo-HSCT治疗儿童AML的适应证、预处理方案及移植后原发疾病复发的预防，形成有中国特色儿童AML造血干细胞移植临床路径及诊疗规范，根据国际最新临床研究成果及国内儿童AML诊治进展及现状，我们制订了本专家共识。

一、儿童AML的诊断及危险度分层

（一）儿童AML的诊断

儿童AML的诊断包含①骨髓细胞形态学包括细胞形态学、细胞化学、组织病理学；②使用多参数流式细胞术进行免疫表型检测；③使用染色体显带技术进行核型分析，必要时行荧光原位杂交（FISH）；④分子学检测融合基因及基因突变；⑤脑脊液检查除外中枢神经系统白血病；⑥影像学检查如B超、CT、MRI或PET-CT等除外其他部位的髓外受累[5]。

（二）儿童AML的预后因素

儿童AML预后因素包括宿主因素、临床特征、细胞遗传学、分子生物学特点及对治疗的反应。宿主因素对预后有重要影响，如小部分儿童AML可能由先天骨髓衰竭性疾病（如范可尼贫血）转化而来或由骨髓增生异常综合征（MDS）进展所致，预后较差。儿童AML治疗反应亦为重要预后因素，如儿童中高危AML诱导化疗后微小残留病（MRD）高水平（流式细胞术检测到MRD≥10^−2^）是复发的独立危险因素[6]。

儿童AML不良预后因素：①有MDS或骨髓增殖性肿瘤（MPN）病史；②治疗相关性/继发性AML。

（三）儿童AML危险度分层

既往儿童AML危险度分层乃是基于细胞形态学及细胞遗传学，其后改进的细胞遗传学技术、流式细胞术、分子生物学技术检测融合基因及靶向测序鉴定出许多具有预后意义的儿童AML生物学标志。一些之前被认为有预后意义的临床及生物学指标（如年龄）被逐渐弃用，而二代测序技术则提供了一些新标志[7]–[9]。目前，我们推荐根据细胞遗传学及分子遗传学定义的儿童AML危险度分层（表1）。

**表1:** 儿童急性髓系白血病（AML）的危险度分层

预后分层	细胞遗传学	分子遗传学
预后良好	t(8;21)(q22;q22)/RUNX1-RUNX1T1； Inv16(p13;q22）/t(16;16) (p13;q22)/CBFB-MYH11	NPM1突变且无FLT3突变或FLT3低等位基因比； CEBPA双突变
预后中等	所有除了预后良好及预后不良的类型	
预后不良	5号、7号染色体单体；5q−；7q−； inv(3)(q21q26.2)或t(3;3)(q21;q26.2)/RPN1-MECOM/RUNX1-MECOM； t(6;9)(p23;q34)/DEK-NUP214； 11p15/NUP98基因重排； t(7;12)(q36;p13)/ETV6 /MNX1[10]–[11]； t(4;11)(q21;q23)/KMT2A-AFF1(AF4)； t(6;11)(q27;q23)/KMT2A-AFDN(AF6)； t(10;11)(p12.3;q23.3)/KMT2A-MLLT10(AF10)；t(10;11)(q12.1;q23.3)/KMT2A-ABI1； t(9;22)(q34;q11.2)/BCR-ABL1； t(16;21)(p11.1;q22.2)/FUS-ERG； Inv16(p13.3q24.3)/CBFA2T3-GLIS2[12]–[13]； 复杂核型（≥3种），但不包括良好核型	FLT3-ITD高等位基因比^a^（NPM1野生型）

注：^a^突变/野生的比值≥0.5为高等位基因比

二、儿童AML的allo-HSCT适应证

儿童AML的移植适应证包括患者的疾病类型、危险度分层及对治疗的反应。

（一）预后良好患儿

1. 第1次完全缓解（CR_1_）期：有经验的移植中心对于RUNX1-RUNX1T1基因阳性儿童AML在两个疗程巩固化疗后骨髓基因下降<3个log（一般基因定量≥0.4％）或强化治疗后基因由阴性转阳性者考虑allo-HSCT[14]–[15]。

2. 第2次及以上完全缓解（≥CR_2_）期：推荐allo-HSCT。

（二）预后中等患儿

1. CR_1_：预后中等患儿建议allo-HSCT[16]，也可根据患儿临床体能结合化疗反应（监测MRD水平）来决定。

2. ≥CR_2_：推荐allo-HSCT。

（三）预后不良患者

推荐所有预后不良儿童AML患者于CR_1_期行allo-HSCT[17]。

（四）转化型AML（t-AML）

t-AML包括治疗相关AML（化疗或放疗后继发AML）和MDS进展而来的AML。

（五）治疗反应不佳患者

标准诱导方案化疗第1疗程结束后骨髓MRD高水平（流式细胞术MRD≥10^−2^）。

（六）经过≥3个诱导化疗无法达到CR的儿童AML患者

经过≥3个诱导化疗无法达到CR的患者，建议allo-HSCT。

（七）儿童AML血液学复发后使用各种化疗方案和（或）免疫治疗手段未获得CR的患者，建议allo-HSCT。

三、儿童AML患者allo-HSCT前的处理

（一）降低移植前肿瘤负荷

儿童中高危AML患者移植效果与肿瘤负荷密切相关[18]–[19]，移植前获得CR、达到MRD阴性患儿可继续巩固1～3个疗程后进行allo-HSCT。若3个疗程标准诱导化疗仍未取得形态学缓解，建议行allo-HSCT、挽救治疗或进入临床试验。

难治/复发AML患者为获血液学缓解，在患儿监护人充分知情同意情况下，可根据临床症状、体征等选用新型靶向/免疫治疗方案（如BCL-2抑制剂+去甲基化药物）联合或不联合常规化疗[20]，也可采用CAR-T细胞治疗[21]、靶向药物（如酪氨酸激酶抑制剂）[22]–[23]。

（二）髓外病灶的处理

1. 中枢神经系统白血病（CNSL）的预防：儿童AML患者移植前应完成至少2～4次鞘内注射。移植后可根据患儿疾病状态及临床体征综合判断CNSL的监测次数及时机并作相应诊治；WBC≥40×10^9^/L、有髓外浸润、AML-M_4_/M_5_、t（8；21）/RUNX1-RUNX1T1、inv（16）患者进仓前争取完成4～6次鞘内注射，移植后鞘内注射1次，若移植前鞘内注射次数不够，可于移植后补齐。

2. 其他髓外病灶的处理：伴髓系肉瘤者建议移植前使用影像学手段（包括PET-CT）评估髓外病灶。若有残余病灶，有条件者可使用局部放疗、手术等手段清除病灶后行allo-HSCT，亦可考虑采用全身放射治疗（TBI）+局部增强照射的预处理方案。

四、造血干细胞来源及供者选择

儿童AML移植物来源优先考虑HLA相合同胞供者；若无同胞HLA相合供者，单倍体相合供者、非血缘全相合供者、脐带血亦可采用。有报道显示对于高危儿童AML，单倍体造血干细胞移植与同胞相合移植相比有更好的无病生存率（DFS）及更低的移植后3年累积复发率[24]。

（一）单倍体相合供者

非体外去T细胞单倍体造血干细胞移植方案（北京方案）推荐儿童AML单倍体相合供者选择：父亲、母亲（≤45岁）、非遗传性母亲抗原（NIMA）不合的同胞、非遗传性父亲抗原（NIPA）不合的同胞及其他旁系亲属[25]。对于拟选择亲缘供者、年龄较小的患者应注意除外是否为遗传易感性髓系肿瘤。

后置环磷酰胺体内去除T细胞（PT-CY）儿童AML单倍体造血干细胞移植方案的供者选择也可参考：父亲、兄弟姐妹、母亲等[26]–[27]。体外去除T细胞单倍体造血干细胞移植治疗儿童恶性血液病，要注意选择供者NK细胞对患者细胞具有同种异体反应性的供者[28]–[29]。

供者特异性抗人类白细胞抗原抗体（DSA）与单倍体造血干细胞移植后植入不良相关。在充分告知儿童监护人的情况下，移植前筛查DSA，若数值小于2 000，无需处理；若DSA为2 000～10 000，可联合加入脐带血辅助，根据各移植中心自身经验，可采用抗CD20单抗、静脉丙种球蛋白、血浆交换及联合方案等干预方法；若数值>10 000，建议更换移植供者[30]–[31]。

（二）脐血造血干细胞移植

脐血是儿童AML患者allo-HSCT较好的造血干细胞来源[32]。在选择脐血时应综合考虑供患者HLA相合程度、脐血总有核细胞数（TNC）、CD34^+^细胞数和DSA。推荐选择脐血的标准：①患者进行高分辨HLA12位点配型，以供受体HLA高分辨配型6个位点（HLA-A、-B、-DR）在中国公共脐血库中初筛，初选脐血以供患者HLA 4～6/6位点相合、脐血冷冻前TNC>2.5×10^7^/kg（患者体重）和CD34^+^细胞>1.5×10^5^/kg（患者体重）；如果6/6位点相合，脐血细胞数可以降低TNC>2.5×10^7^/kg（患者体重）和CD34^+^细胞>1.2×10^5^/kg（患者体重）。②初筛后的脐血进行小管复苏，要求TNC活力>80％，CD34^+^细胞活力>90％，CFU-GM集落生长良好。③确认脐血需要满足以上条件，同时建议供患者HLA高分辨配型10个位点（HLA-A、-B、-Cw、-DR、-DQ）达到4/6、5/8、6/10位点相合。④如果检出DSA，可重新选择脐血[33]。

五、预处理方案的选择

（一）TBI与非TBI的选择

在allo-HSCT治疗儿童AML的预处理方案中，TBI预处理方案与非TBI方案临床结局无差异；考虑到TBI对儿童具有长期毒性，建议选择以BuCy为主的预处理方案[34]–[36]。若使用非TBI方案时，建议根据白消安血药浓度调整其剂量。

（二）单倍体造血干细胞移植方案

非体外去T细胞单倍体造血干细胞移植方案（北京方案，表2）应用较广[37]–[38]，在儿童AML中具有良好的疗效[15]–[16],[24]。

**表2:** 改良的儿童急性髓系白血病（AML）异基因造血干细胞移植清髓预处理方案

预处理方案	适用移植类型	具体方案
mBuCy	Sib-HSCT	羟基脲80 mg/kg（分2次），−10 d；阿糖胞苷2 g/m^2^，−9 d；白消安9.6 mg/kg（静脉），−8 ~ −6 d；环磷酰胺3.6 g/m^2^，−5 d、−4 d；MeCCNU 250 mg/m^2^（口服），−3 d
mCy/TBI	Sib-HSCT	单次TBI 770 cGy，−6 d；环磷酰胺3.6 g/m^2^，−5 d、−4 d；MeCCNU 250 mg/m^2^，−3 d
mBuCy+ATG	UD-HSCT、CBSCT、haplo-HSCT	阿糖胞苷4 ~ 8 g/m^2^，−10 d、−9 d；白消安9.6 mg/kg（静脉），−8 ~ −6 d；环磷酰胺3.6 g/m^2^，−5 d、−4 d；rATG 10 mg/kg，−5 ~ −2 d或ATG-F 40 mg/kg，−5 ~ −2 d
mCy/TBI+ATG	UD-HSCT、CBSCT、haplo-HSCT	单次TBI 770 cGy，−6 d；环磷酰胺3.6 g/m^2^，−5 d、−4 d；MeCCNU 250 mg/m^2^，−3 d；rATG 10 mg/kg，−5 ~ −2 d或ATG-F 40 mg/kg，−5 ~ −2 d

注：TBI：全身放射治疗；MeCCNU：甲环亚硝脲；ATG：抗胸腺细胞球蛋白；rATG：兔抗人胸腺细胞球蛋白；ATG-F：抗人T细胞兔免疫球蛋白；Sib-HSCT：同胞全相合造血干细胞移植；CBSCT：脐血造血干细胞移植；haplo-HSCT：单倍体造血干细胞移植；UD-HSCT：非亲缘造血干细胞移植

（三）减低毒性预处理

预处理方案相关毒性是限制高剂量造血干细胞移植预处理应用的主要因素。为尽量减少预处理毒性，利用移植物抗肿瘤效应，各种减低强度预处理方案（RIC）被提出，但均应符合以下标准：①TBI单次给予，剂量≤5 Gy；或TBI分次给予，剂量应≤8 Gy；②白消安<9 mg/kg；③美法仑<140 mg/m^2^；④塞替派≤10 mg/kg。对于无法耐受标准预处理方案的儿童AML可选用RIC方案。建议RIC方案用于不能耐受清髓性预处理方案的患儿，如唐氏综合征相关AML移植以及二次移植等[39]–[40]。

（四）增强预处理

未达到缓解进行挽救移植的患儿、高危AML可考虑接受增强预处理方案。增强预处理方案一般在经典方案基础上增加放疗剂量或增加化疗药物，常用地西他滨、美法仑、氟达拉滨、依托泊苷及塞替哌等。

六、儿童AML移植后复发/MRD阳性的预防及干预

（一）移植后复发的预防

1. 预防性供者淋巴细胞输注（DLI）：推荐移植前未达到血液学缓解或CR3的儿童AML患者进行预防性DLI。同胞全相合造血干细胞移植患者在移植后30 d左右，单倍体造血干细胞移植患者在移植后45 d～60 d进行预防性DLI[41]。DLI前无需化疗，回输动员后的外周血干细胞（单个核细胞1×10^8^/kg、CD3^+^细胞1×10^6^/kg～1×10^7^/kg）。预防性DLI后原有基础免疫抑制剂维持有效浓度继续应用至少6周，若无GVHD则8周减停。若无GVHD，6个月后可重复回输1次。

2. 靶向药物的预防性应用：①FLT3-ITD阳性儿童AML在移植后建议口服索拉非尼预防复发[42]。若患儿在移植后30 d左右，无活动性GVHD及活动性感染，推荐口服索拉非尼直至移植后6个月～2年。使用过程中监测血象、肝肾功能。若中性粒细胞计数小于1000，血小板计数小于5万，可暂停索拉非尼；若为FLT3-TKD突变阳性，可考虑口服米哚妥林[43]。②去甲基化药物：儿童高危AML若无合适靶向药物，可考虑使用去甲基化药物预防复发，阿扎胞苷及地西他滨均可用于预防复发[44]–[45]。

（二）移植后MRD阳性的干预

可根据各移植中心经验对移植后AML患儿进行定期骨髓评估及病情监测。定期监测骨髓项目包括骨髓涂片、流式细胞术MRD、融合基因或WT1基因、FISH（供患者性别不同或有特殊染色体标志的患者）、外周血DNA指纹图。MRD阳性定义为流式细胞术MRD间隔2周连续2次阳性（≥10^−3^）或特异性融合基因（RUNX1-RUNX1T1、CBFβ-MYH11、MLL基因）转为阳性（一般RT-PCR敏感度为10^−5^～10^−6^）。随访中一旦确定MRD阳性，应立即减/停免疫抑制剂，若减/停免疫抑制剂无效，应立即给予干预[46]。

1. 干扰素：移植后MRD阳性儿童AML患者停用环孢素A 2周后复查，若仍为阳性，可给予干扰素100 000 U/kg（最高300 000 U/kg）每周2次皮下注射，1个月后复查骨髓评估疗效[47]–[48]。若应用干扰素期间出现明显皮疹、腹泻，应立即停药，由移植医师判断是否继续使用。

2. DLI：DLI是儿童AML血液学复发后最重要的免疫治疗[49]–[50]。供者T淋巴细胞免疫耗竭是移植后白血病复发原因之一，而DLI有可能逆转免疫耗竭。改良的供者淋巴细胞输注（mDLI）使用粒细胞集落刺激因子（G-CSF）动员的外周血前体细胞，并在输注后予短程免疫抑制剂预防DLI相关GVHD。建议儿童AML患者MRD阳性且干扰素无效或流式细胞术MRD及特异性融合基因检测同时阳性者，可考虑使用化疗序贯mDLI。mDLI前化疗可使用之前有效或未曾使用过的化疗方案，例如HAA方案（高三尖杉酯碱+阿糖胞苷+阿克拉霉素）。环孢素A于mDLI前1 d开始应用，建议同胞全相合造血干细胞移植患者mDLI后4～6周、单倍体造血干细胞移植患者mDLI后6～8周减停环孢素A。若mDLI后MRD阴性且未发生GVHD，可考虑继续干扰素治疗[51]。

（三）移植后复发的治疗

儿童AML移植后复发后推荐如下评估项目：①完善免疫分型、融合基因、染色体、HLA-loss等检查，除外移植后继发白血病；②建议做二代测序检测白血病相关基因及突变，寻找可能有效的靶向药物；③完善免疫分型，寻找可能的CAR-T细胞治疗靶点（如CD33）；④应使用影像学（如PET-CT）评估有无髓外浸润。

1. 治疗性mDLI：化疗方案的选择及回输剂量可参考移植后MRD阳性患者的化疗序贯mDLI方案。患儿若检出相应靶点，化疗方案中可加用相关靶向药物或去甲基化药物。若mDLI后达到CR，可考虑每3～6个月进行1次治疗性DLI，为期1年。

2. CAR-T细胞治疗：使用以CD33为靶点的CAR-T细胞治疗AML目前尚属于临床试验阶段[52]–[53]。治疗后往往血象较差，常需进行二次移植。

3. 二次移植：患儿血液学复发、经化疗序贯mDLI后再次达到CR，其后可根据患者身体状况、个人意愿决定是否进行二次移植。若为单倍体造血干细胞移植，应根据是否存在HLA-loss来选择供者。二次移植方案亦应根据疾病类型、患儿身体状态、脏器功能等综合考虑[54]–[55]。

4. 移植后髓外复发的治疗：儿童AML的髓外复发包括CNSL、睾丸及其他局部病灶复发，可单独发生，也可伴有骨髓复发[56]。若出现CNSL或睾丸复发，可在局部放疗后行DLI预防骨髓复发；其他局部病灶可在评估病情后行化疗或局部放疗，随后行DLI预防血液学复发。allo-HSCT是儿童AML重要的巩固治疗手段，需要精准的危险度分层、适时的造血干细胞移植以及移植后合理的预防复发、及时的抢先干预及规范诊疗，如此才能进一步提高我国儿童AML患者的长期生存率。
